# Enhancing surgical precision in early‐stage non‐small cell lung cancer: A novel approach through temporary pulmonary vascular occlusion

**DOI:** 10.1111/1759-7714.15388

**Published:** 2024-06-04

**Authors:** Yan Zhao, Bin You, Hui Li

**Affiliations:** ^1^ Department of Thoracic Surgery Beijing Institute of Respiratory Medicine and Beijing Chao‐Yang Hospital, Capital Medical University Beijing China

**Keywords:** fluorescence thoracoscopy, lung cancer, minimally invasive surgery, sublobectomy

## Abstract

**Background:**

To evaluate a novel intraoperative localization technique utilizing temporary pulmonary arteriovenous occlusion for enhancing the precision of sublobar resections in early‐stage NSCLC.

**Methods:**

Conducted from January to November 2023, this study involved 140 patients. During the surgery, key pulmonary vessels were identified using preoperative three‐dimensional (3D) imaging and temporarily occluded with noninvasive clamps to isolate the target lung segment. Following vascular occlusion, indocyanine green (ICG) was administered intravenously to precisely delineate the resection margins. After visually confirming the marked areas, the clamps were released, and a targeted partial resection was performed on the delineated segment. Surgical data, including operation times, surgical margins, and hospitalization costs, were collected and compared with those from a historical control group of 110 patients who underwent traditional pulmonary wedge resections.

**Results:**

In the study group, the median surgical margin achieved was 16 mm, which was statistically significant compared to 15 mm in the control group (*p* < 0.05). Operation times were reduced to an average of 58.43 ± 12.962 min, showing a decrease from the control group's average of 69.50 ± 17.544 min (*p* < 0.05). Hospitalization costs were also lower, averaging $4772.98 ± 624.339 for the study group versus $5161.34 ± 856.336 for the control group (*p* < 0.05). Patient safety was maintained with no increase in surgical complications.

**Conclusion:**

The technique, leveraging temporary pulmonary arteriovenous occlusion, offered a significant advancement in the surgical treatment of peripheral early‐stage NSCLC. It reduced operation time and lowered overall surgical costs. This method represented a promising alternative to traditional surgical approaches.

## Graphical abstract

This study introduces a novel intraoperative localization method utilizing temporary pulmonary arteriovenous occlusion, improving precision in sublobar resections for early‐stage NSCLC. The technique enhances margin accuracy, reduces surgery time and hospitalization costs, and demonstrates clinical feasibility and benefits over traditional methods.

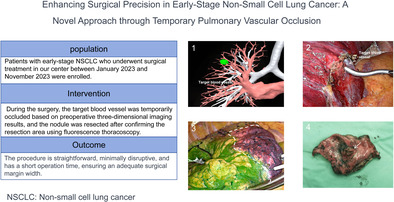

## INTRODUCTION

Lung cancer is the leading cause of cancer‐related mortality worldwide. Surgical resection plays a crucial role in improving survival rates for patients with early‐stage non‐small cell lung cancer (NSCLC). Traditionally, lobectomy has been considered the gold standard for such resections. However, recent advancements in surgical strategies and outcomes research have prompted a reassessment of this approach, particularly for tumors characterized by ground‐glass opacities (GGOs).

Sublobar resections, including segmentectomy and wedge resection, have gained increasing acceptance as a potentially preferable option, especially for peripheral lung tumors ≤2 cm with a predominant GGO component. The JCOG0804 trial and other studies within the JCOG framework have been instrumental in this shift. These studies suggest that sublobar resections, when performed with adequate surgical margins, offer comparable oncological outcomes with lobectomy for certain NSCLC cases, with significantly reduced postoperative morbidity and better preservation of pulmonary function.^1^, ^2^, ^3^

The precision in localizing small GGO nodules, which are often not palpable during surgery, is crucial for successful sublobar resections. Traditional preoperative computed tomography (CT)‐guided needle localization, while direct, carries risks such as bleeding, pneumothorax, and sometimes inaccurate nodule marking, potentially complicating surgical outcomes. Exclusively relying on a surgeon's anatomical knowledge for intraoperative localization can lead to inconsistent results, including the unnecessary removal of larger lung segments or missed lesions.

In response to these challenges, our institution has developed a novel intraoperative localization technique aimed at enhancing the precision and efficiency of sublobar resections for peripheral early‐stage NSCLC. This innovative approach utilizes temporary regional occlusion of the pulmonary artery or vein, enabling the accurate localization and resection of small pulmonary nodules. An evaluation has been conducted to validate the safety and effectiveness of this technique among a selected cohort of patients.

## METHODS

### Participants

This prospective, single‐arm study was designed to evaluate the clinical outcomes of patients diagnosed with early‐stage NSCLC undergoing surgical treatment at our institution from January 2023 to November 2023. To ensure the precision of nodule localization and the assessment of adjacent pulmonary vessels, all participants underwent detailed preoperative three‐dimensional (3D) imaging. This imaging technique facilitated the accurate identification of the target nodules and a comprehensive understanding of the vascular architecture, which is critical for planning the extent of resection and minimizing intraoperative risks.

The surgical protocol was standardized across all cases by a dedicated surgical team, incorporating the strategic temporary occlusion of specific branches of the pulmonary artery or vein. This was followed by an executed sublobectomy.

Comparative analysis was conducted against a cohort of historical controls. These controls consisted of patients treated with pulmonary wedge resection for peripheral early‐stage NSCLC under similar conditions and by the same surgical team. The selection of historical control cases was specifically from the period of July 2022 to December 2022. This comparison aimed to establish a benchmark for evaluating the innovative surgical technique's effectiveness and safety.

Ethical approval for this study was granted by the Ethics Committee of our hospital, affirming compliance with both institutional guidelines and the ethical standards. The importance of patient confidentiality and data protection was paramount throughout the research process. In line with ethical research practices, all participants were provided with an explanation of the surgical procedures involved by the attending physician. Informed consent was obtained from each patient, with documentation of consent secured prior to any surgical intervention.

### Inclusion criteria

1. Chest CT should meet the following criteria:
  - High suspicion of NSCLC based on imaging examination.
  - Nodules should be located in the outer half of the lung.
  - The primary lesion should be subsolid or a pure ground glass nodule ≤2 cm in diameter, with a solid component of less than 25%.
  - There should be a single tumor or multiple tumors, with other lesions besides the primary lesion not having undergone special treatment within 1 year.
2. Preoperative three‐dimensional computed tomography bronchial angiography (3D‐CTBA) results should indicate that there were branches of pulmonary artery or vein passing through the nodule or within 1 cm of the nodule. Preoperative planning of vascular branches that were suitable for dissection and temporary occlusion was required.

### Exclusion criteria

1. The patient was found to have an allergy to iodine.
2. Inadequate cardiopulmonary function to tolerate surgical treatment.

### Preoperative planning

Upon the patient's admission, a comprehensive evaluation process was initiated, beginning with a high‐resolution chest CT scan to confirm the lesion's eligibility for surgical intervention. This initial step was crucial for detailed visualization of the lesion and its spatial relationship with surrounding anatomical structures. Concurrently, cardiopulmonary assessments were conducted to identify any potential surgical contraindications, ensuring that patients were fit for the planned surgical procedure.

Following these preliminary assessments, the CT data were imported into software designed for 3D reconstruction. This technology allowed for the creation of a detailed 3D model of the patient's bronchial tree, arterial and venous networks, and the lesion itself.

Particular attention was paid to identifying reference vessels—those arterial or venous branches in close proximity to the lesion. These vessels were earmarked as critical landmarks for intraoperative navigation. The 3D model facilitated an evaluation of the feasibility of dissecting and temporarily occluding these vessels during surgery.

### Surgical approach

All patients who met the eligibility criteria were subjected to double‐lumen tracheal intubation under general anesthesia, facilitating single‐lung ventilation while positioned laterally on the healthy side. The surgical entry was achieved via a fluorescence thoracoscopic approach, utilizing a single 3 cm incision located at the fourth or fifth intercostal space along the anterior axillary line, dictated by the nodule's precise location. An auxiliary observation port was established at the seventh or eighth intercostal space on the midaxillary line. To assist in precise intraoperative navigation, real‐time 3D imaging was rendered on a mobile device placed adjacent to the thoracoscopic screen.

The surgical procedure commenced by first identifying the branches of the primary pulmonary arteries or veins that were closely associated with the nodules, as highlighted in the preoperative 3D imaging. These vascular branches were carefully isolated while preserving the integrity of the main vascular trunks. Noninvasive vascular clamps were then applied to these specific branches to achieve temporary occlusion. For visual differentiation of tissue perfusion, a solution containing 25 mg of indocyanine green (ICG) in 10 mL of diluent was prepared, from which 1 mL was intravenously administered following occlusion. Near‐infrared fluorescence thoracoscopy, set to fluorescence mode, delineated the nonpigmented regions indicative of blood flow occlusion from the green‐pigmented, preserved lung tissue, thereby demarcating clear boundaries for excision. The boundaries illuminated by fluorescence were subsequently marked on the visceral pleura using electrocoagulation, ahead of excision with an endoscopic stapler. The duration for which each patient's fluorescence boundary remained visible was recorded.

Concomitantly, a biopsy of the lymph nodes within the tumor's lymphatic drainage area was conducted, with specimens sent for rapid pathological examination. Should these examinations reveal tumor invasion, the patient would be removed from the study, and a lobectomy would be pursued instead.

The precise location of the lesion within the excised specimen was identified for every patient. The minimum distance between the lesion and the excision margin was measured, followed by the dispatch of the specimen for pathological review. In instances where malignancy was confirmed, a detailed dissection or biopsy of the pulmonary hilar and mediastinal lymph nodes was undertaken. Any air leakage detected at the resection margin stump during the air leak test necessitated suture reinforcement, further secured with the application of polyglycolic acid felt. A 24F closed thoracic drainage tube was installed thereafter.

In the postoperative phase, chest drainage was monitored, with removal dependent on satisfactory pulmonary re‐expansion, the absence of air leakage, and the maintenance of drainage volume below 300 mL within a 24‐h period. Fulfillment of these criteria initiated the protocols for patient discharge.

For lung wedge resections, localization and selection methods varied based on the operating surgeon's preferences. Specifically, a portion of patients underwent preoperative CT‐guided lung puncture for nodule localization. Alternatively, some patients were subjected to intraoperative wedge resections, with the surgeon utilizing CT imaging and personal experience to identify and excise the nodules. Case selection and operative details were derived from patient medical and surgical records, enabling a statistical analysis of the procedures.

### Data collection and outcome measures

Data collection for the cohort was conducted prospectively, encompassing a wide range of parameters such as demographic details, tumor characteristics, operative data, postoperative outcomes, and follow‐up findings. Specifically, the cost of hospitalization was calculated to provide a comprehensive analysis of the economic impact of the treatment modalities explored. This included all charges incurred during the hospital stay from admission to discharge, covering the surgical procedure, room charges, medication, use of medical facilities such as the operating and recovery rooms, and any additional therapeutic interventions or complications that incurred costs. These data were sourced directly from the hospital's billing department, which provided itemized billing statements for each patient, ensuring the accuracy and relevance of the financial data.

In addition to financial data, the method for measuring surgical margins was standardized across both the experimental and historical controls. Immediately following the excision of the specimen, surgical margins were measured by an assistant using a ruler. These measurements were consistently recorded in the operative records for each patient, ensuring uniformity in data collection. To facilitate a robust comparative analysis, historical control data were extracted from medical records. These controls were selected based on identical eligibility and exclusion criteria applied to patients who underwent wedge resection for peripheral early‐stage NSCLC. Special attention was dedicated to aligning outcome measures between the study cohort and historical controls, ensuring direct comparability.

### Statistical analysis

Perioperative data were collected, including demographics, nodule location, surgical details, intraoperative complications, specimen tumor size, resection margin distance, pathology, postoperative recovery, and hospital stay duration. Follow‐up at 1 and 3 months post‐surgery included symptom assessment and chest CT scans. Data were analyzed using SPSS19.0, with measurement data presented as mean ± standard deviation or median (quartile), and count data as n (percentage). A Mann–Whitney U test was applied for data comparison.

## RESULTS

In the study, we initially identified 144 patients whose CT imaging and subsequent 3D imaging results met the enrollment criteria. However, four patients with a history of iodine contrast allergy were excluded from the group. Consequently, 140 patients underwent sublobar resections utilizing temporary pulmonary arteriovenous occlusion to treat pulmonary nodules. This group comprised 71 male and 69 female patients, with an average age of 58.06 ± 12.773 years, spanning from 34 to 80 years. Preoperative CT imaging revealed that all tumors were subsolid or pure ground glass nodules, with less than 25% of the mass being solid. The median tumor size, with interquartile intervals, was measured at 10 (8, 13) mm.

Notably, no patient required a shift to thoracotomy due to bleeding or organ injury. Complete resection of the lesions was successfully performed in all cases, avoiding any extended resections for leftover lesions or insufficient margins. The median measurements for surgical margin distance and the average duration of operation were 16 mm (13, 18) and 58.43 ± 12.962 min, respectively (Table 1).

**TABLE 1:** Baseline characteristics of eligible patients.

Factors	Data (%) or mean ± SD or median (quartile)
Sex	
Male	71 (50.7%)
Female	69 (49.3%)
Age (year)	58.06 ± 12.773
Pathology	
AIS	57 (60%)
MIA	50 (33.33%)
AAH	28 (6.67%)
IA	5 (3.6%)
Tumor diameter (mm)	10 (8, 13)
Margin distance (mm)	16 (13, 18)
Duration of surgery (min)	58.43 ± 12.962
Duration of drainage (days)	2 (2, 3)
Postoperative hospital stay (days)	3 (2, 3)
Drainage volume on POD 1 (mL)	150 (100, 200)
Drainage volume on POD 2 (mL)	150 (100, 200)
Hospitalization expenses (USD)	4772.98 ± 624.339

Abbreviations: AAH, atypical adenomatous hyperplasia; AIS, adenocarcinoma in situ; IA, invasive adenocarcinoma; MIA, minimally invasive adenocarcinoma; POD, postoperative day; USD, US dollar.

**FIGURE 1:**
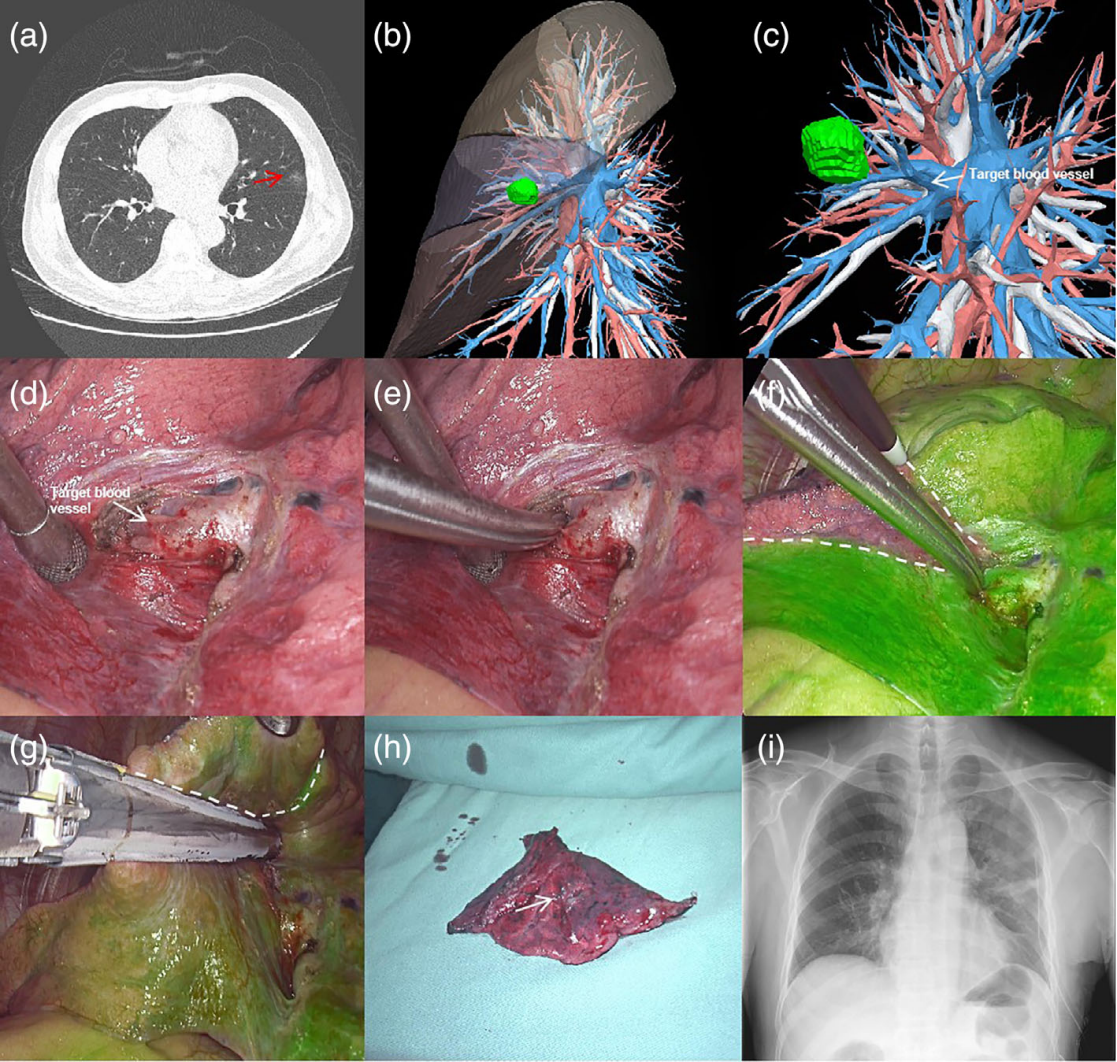
(a) Chest computed tomography (CT) examination revealed the precise localization of the nodule in the lingual segment of the upper lobe of the left lung, situated in close proximity to the pleura. (b,c) Based on preoperative three‐dimensional computed tomography bronchial angiography (3D‐CTBA) technology, the target blood vessel within the region of the nodule was precisely identified and delineated. (d,e) During the surgical procedure, the target vessel was exposed and temporarily occluded using a noninvasive vascular clamp. (f,g) Following intravenous injection of indocyanine green (ICG), the target area did not exhibit any staining, while the surrounding lung parenchyma displayed a green fluorescence. Subsequently, the target area was successfully excised using endoscopic staplers. (h,i) The resection margin of the specimen met the criteria for tumor resection, ensuring adequate removal of the tumor. A postoperative chest x‐ray demonstrated a satisfactory recovery outcome, indicating favorable progress following the surgical procedure.

Temporary occlusion involved 57 arterial and 83 venous cases. Some of the occluded vessels, being thin and distally located, were challenging to identify precisely; thus, they were classified according to their general location within the pulmonary segments (Table 2).

**TABLE 2:** Distribution of nodule locations for temporary pulmonary vessel occlusion.

Segment (lobe)	Arterial occlusion	Venous occlusion
S4 (left upper)	7^a^	1
S1 + 2 (left upper)	10	‐
S6 (left lower)	9	‐
S2 (right upper)	10	‐
S5 (right middle)	2	9
S6 (right lower)	10	‐
S8 (right lower)	9	‐
S3 (right upper)	‐	15^b^
S9 (right lower)	‐	6
S3 (left upper)	‐	9
S10 (left lower)	‐	16
Between S6 and S10 (left lower)	‐	15
Between S9 and S10 (left lower)	‐	12

^a^ The specific surgical procedure is shown in Figure 1.

^b^ The specific surgical procedure is shown in Figure 2.

**FIGURE 2:**
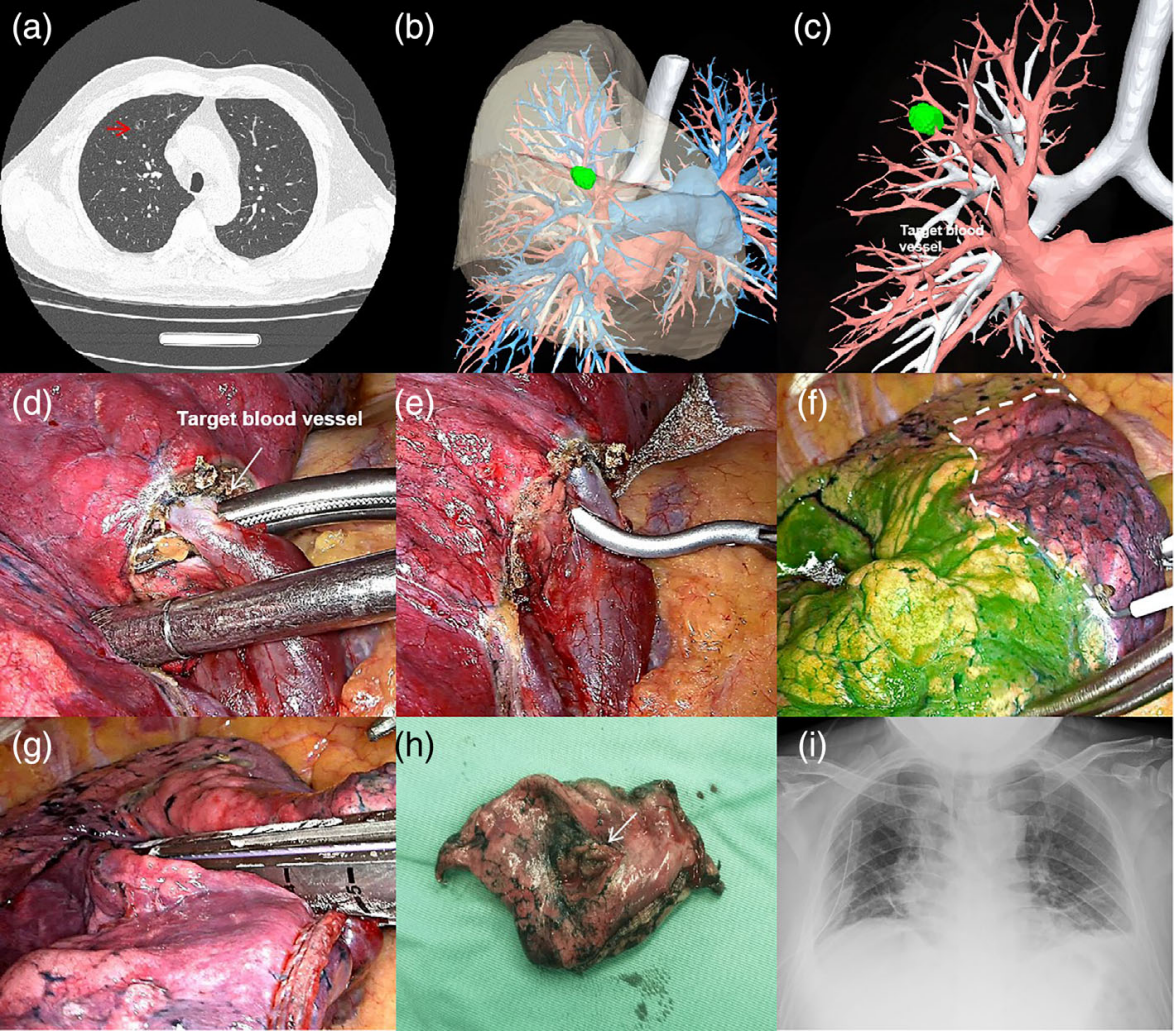
(a) Chest computed tomography (CT) examination revealed the precise localization of the nodule in the anterior segment of the right upper lobe of the lung. (b,c) The target vessel within the region where the nodules were located was identified and marked using three‐dimensional computed tomography bronchial angiography (3D‐CTBA) prior to the surgical procedure. (d,e) During the surgical procedure, the target vessel was dissected and temporarily occluded using noninvasive vascular clamps. (f,g) Following the intravenous injection of indocyanine green (ICG), a well‐defined resection boundary was observed. Subsequently, the target area was completely resected using endoscopic staplers, ensuring thorough removal of the intended tissue. (h,i) The nodule was successfully excised, and the resection margin was deemed adequate. Postoperative chest radiographs showed favorable recovery, indicating a positive outcome following the surgical procedure.

The analysis revealed a significant difference in the duration of surgical boundary maintenance between the temporary arterial and venous occlusion groups (150 s [136.5, 168] vs. 41 s [34, 46], *p* < 0.001) (Table 3). This highlights the differential impact of arterial versus venous occlusion on the surgical procedure's duration.

**TABLE 3:** Comparative analysis of surgical outcomes in NSCLC patients undergoing temporary pulmonary artery versus vein occlusion.

	Artery (57)	Vein (83)	*Z*	*p*‐value
Margin distance (mm)	16 (14, 19)	15 (13, 18)	−0.554	0.306
Duration of surgery (min)	60 (50, 65)	60 (50, 70)	−0.332	0.740
Duration of drainage (days)	2 (2, 3)	2 (2, 3)	−0.927	0.354
Postoperative hospital stay (days)	3 (2, 3)	3 (2, 3)	−0.021	0.984
Drainage volume on POD 1 (mL)	150 (100, 200)	150 (100, 200)	−0.340	0.734
Drainage volume on POD 2 (mL)	150 (100, 200)	162.5 (100, 200)	−0.163	0.871
Hospitalization expenses (USD)	4685.32 ± 635.194	4833.19 ± 613.355	−1.487	0.137
Duration of boundary (s)	150 (136.5, 168)	41 (34, 46)	−10.037	0.000

Abbreviations: NSCLC, non‐small cell lung cancer; POD, postoperative day; USD, US dollar.

The postoperative period for all 140 patients was marked by successful recovery, free of complications such as atelectasis, pulmonary infection, hemoptysis. There were no reports of perioperative mortality or hospital readmissions. Notably, one patient experienced prolonged air leakage postoperatively, with drainage extending to 8 days. The duration of postoperative drainage averaged 2 days (2, 3), and the length of the hospital stay following surgery was typically 3 days (2, 3). The volume of chest drainage recorded on the first‐ and second‐days post‐operation was 150 mL (100, 200), respectively. The average cost for hospitalization amounted to 4772.98 ± 624.339 USD. Pathological examinations post‐surgery identified 57 cases of adenocarcinoma in situ (AIS), 50 cases of minimally invasive adenocarcinoma (MIA), 28 cases of atypical adenomatous hyperplasia (AAH), and five cases of invasive adenocarcinoma (IA). Importantly, there were no findings of mediastinal lymph node metastasis in any of the patients.

During the study period, a total of 208 patients who met the criteria for pulmonary nodules were screened. Surgical interventions were performed as follows: 110 pulmonary wedge resections, 76 segmental resections, and 22 lobectomies. The focus of this analysis is on the 110 patients who underwent pulmonary wedge resections, as they were directly comparable with our experimental cohort. This group comprised 43 males and 67 females, averaging 56.72 ± 11.36 years in age. Of these, 48 cases utilized CT‐guided localization during surgery, resulting in five instances of pneumothorax and four cases of puncture bleeding from the localization procedure. These patients immediately underwent surgery for excision of the targeted nodule following localization, with another 62 cases undergoing wedge resections based directly on the surgeon's experience and the anatomical location. In 13 instances, an additional extended resection was necessary for clear tumor identification during the initial wedge resection. The median measurements for surgical margin distance were 15 mm (10, 20), showing statistically significant differences when compared with the experimental group (*p* < 0.05). The average surgery duration in the control group was longer than that observed in the experimental group, with an average of 69.50 ± 17.544 min (*p* < 0.05). Similarly, hospitalization costs were higher in the control group, averaging at 5161.34 ± 856.336 USD, indicating statistically significant differences when compared to the experimental group (*p* < 0.05) (Table 4). Although the duration of chest tube placement and hospital stay showed no significant variance, the control group exhibited two cases of persistent air leaks, lasting 7 and 9 days, respectively. A 3‐month follow‐up indicated an absence of postoperative complications such as lung infections, pleural effusion, or hemoptysis within the wedge resection group.

**TABLE 4:** Comparative analysis of surgical outcomes between the treatment and control group.

Outcome measures	Treatment group	Control group	*Z*	*p*‐value
Margin distance (mm)	16 (13, 18)	15 (10, 20)	−2.403	0.016
Duration of surgery (min)	58.43 ± 12.962	69.50 ± 17.544	−4.990	0.000
Duration of drainage (days)	2 (2, 3)	2 (2, 3)	−1.935	0.053
Hospitalization expenses (USD)	4772.98 ± 624.339	5161.34 ± 856.336	−3.570	0.000

Abbreviation: USD, US dollar.

## DISCUSSION

This study introduced a novel approach for real‐time determination of the resection area during surgery for early‐stage NSCLC, leveraging the precision of preoperative 3D imaging combined with intraoperative near‐infrared fluorescence imaging using ICG. Unlike traditional methods, such as wedge resection that relies on CT‐guided percutaneous puncture for preoperative localization, our technique streamlined the process by identifying the pulmonary artery or vein within the nodule area and temporarily occluding these vessels to demarcate the resection boundaries accurately. This simplified sublobectomy method offers several advantages, including the avoidance of complications commonly associated with preoperative puncture, such as pain, anxiety, pneumothorax, pulmonary hemorrhage, and procedural errors arising from such punctures.^4^, ^5^ By relying on advanced imaging techniques, the need for invasive procedures can be minimized, reducing patient discomfort and potential risks.

The accuracy in identifying the extent of resection was crucial for minimizing the unnecessary removal of lung tissue and avoiding inadequate resection margins, which could lead to residual nodules and postoperative complications such as air leakage, atelectasis, hemoptysis, and reimplantation of the thoracic drainage tube.^6^ The pulmonary blood vessels, with their fixed position despite lung collapse or expansion, proved to be a more accurate method for nodule localization compared to other techniques. Our approach involves temporary, rather than complete, occlusion of blood vessels. Once the resection boundaries were determined, occlusion clamps were released to restore blood circulation before the excision of the target area, minimizing the risk of local ischemia or congestion and preserving functional lung tissue.

The comparative analysis between our study group, which employed temporary pulmonary arteriovenous occlusion localization, and the historical control group, who underwent traditional surgery, elucidated notable advancements in surgical pathology margin length, operation duration, and overall surgical expenditure. The disparity's foundation was attributed to the enhanced anatomical precision provided by our method, particularly beneficial for early‐stage lesions predominantly composed of GGOs that were imperceptible through palpation during surgery. This precision not only ensured superior margin integrity compared to traditional wedge resections but also eliminated the need for repetitive nodule palpation. Consequently, this led to reduced operation time and mitigated the risks associated with more extensive resections or even complete lobectomies, resulting in decreased overall surgical costs.

Building upon this foundation of improved anatomical precision, the innovative aspects of our technique and their implications for surgical practice are significant. The concept of vascular‐centered resection, diverging from conventional bronchus‐centered preoperative planning, allowed for resection margins that more closely aligned with tumor‐centered excision principles, offering flexibility in resection strategies and potentially preserving lung function by avoiding excessive tissue removal. The marked vessels, either intra‐ or intersegmental and classified as artery or vein, provided flexibility in resection strategies, especially for nodules located between segments.

The innovation did not stop with just the conceptual shift, it extended into the practical application of these concepts, as demonstrated by our novel use of venous occlusion.^7^ We observed that clear localization boundaries could still be visualized through temporary obstruction of pulmonary vein branches in the nodule area, ensuring precise positioning and acceptable tumor margins. This phenomenon, likely due to the transient interruption of local blood flow circulation affecting ICG delivery, suggested that both arteries and veins were suitable for temporary occlusion to determine the extent of resection. This provided surgeons with more options based on the anatomical characteristics of different lung segments and nodule locations during preoperative planning. For instance, it may have been more convenient to initially block the superficial pulmonary veins in specific lung segments, such as the anterior upper lung.

Expanding on the versatility of our technique, we further explored its implications for enhancing the safety and efficacy of pulmonary segmentectomy. Several studies have proposed using pulmonary circulation occlusion to identify the intersegmental plane during pulmonary segmentectomy.^8^, ^9^, ^10^ By selectively blocking segmental arteries or veins that supply the nodules, the boundary between oxygen‐rich and hypoxic areas could be visualized using the inflation‐collapse method. In the design of a temporary pulmonary arteriovenous occlusion localization method, ICG was selected as the marker instead of relying solely on dilatation and collapse. This decision was primarily driven by the potential risks associated with blood vessel tearing and bleeding during pulmonary dilation when using blocking instruments like the endoscopic vascular bulldog clamps to occlude blood vessels. Consequently, to enhance the safety of the surgical procedure and provide enhanced surgical options based on anatomical characteristics, the intravenous administration of ICG was adopted to accurately demarcate the resection range.

The integration of near‐infrared fluorescence technology, a cornerstone of our approach, represents a significant advance in the field of pulmonary surgery, opening new avenues for surgical precision and patient care.^11^, ^12^, ^13^, ^14^ However, the majority of studies have not reported on the development of ICG after pulmonary vein occlusion. The novel approach presented in this study proposed a single temporary blocking method for the pulmonary circulation, which was not only applicable for temporary occlusion of the pulmonary artery but also enabled effective occlusion of certain pulmonary veins traversing the nodule. This approach significantly broadens the range of operative adaptability, thus offering enhanced surgical options. Although the study results indicated that the display time of the boundary in the venous temporary block group was significantly shorter than that in the arterial temporary block group, it was still sufficient to delineate the boundary before it vanished.

Previous studies recommended a dosage of 0.5 mg/kg for ICG injection to determine intersegmental planes in pulmonary segmentectomy.^12^, ^15^ In this study, regardless of patient weight, a uniform dosage of 2.5 mg of ICG was used, which proved to be sufficient for visualizing clear resection boundaries without any ICG‐related complications. Multiple studies have indicated that ICG is not suitable for patients with poor liver function, ICG or iodine allergies.^16^, ^17^, ^18^ The toxicity reaction to ICG is concentration‐dependent, with a significant increase in allergic reactions when the concentration exceeds 5 mg/kg.^19^ Therefore, strict control of ICG injection volume was necessary during the surgical procedure.

The injection rate was also a crucial aspect of the temporary pulmonary circulation occlusion localization method. Previous research on fluorescence‐assisted thoracoscopic segmentectomy often required rapid intravenous injection of ICG. While this approach quickly established a distinct intersegmental border, the staining duration of the fluorescent dye was brief. Additionally, due to the distribution of vascular branches and collateral circulation, rapid intravenous injection may accelerate the distribution of ICG throughout the entire lung lobe. This effect was particularly evident during isolated pulmonary vein temporary occlusion localization. With fast injection rates, the resection area gradually turned green within a few seconds, resulting in blurred boundaries. To prolong the staining duration, it was recommended to slowly administer ICG through intravenous injection after temporary vessel occlusion, while controlling the infusion rate. This allowed ICG to gradually distribute within the accessible circulation of the lung vasculature. This approach not only provided a clear resection range but also prolonged the duration of staining, facilitating the surgeon in marking the resection boundaries.

In our clinical observations, early‐stage NSCLC patients underwent surgical procedures and were subject to detailed, short‐term postoperative follow‐ups. Over the course of several months, these patients exhibited no significant complications such as pulmonary infections, atelectasis, or hemoptysis, thus affirming the effectiveness and safety of our surgical approach. It is important to highlight that all participants in this study predominantly presented with GGOs, indicative of very early‐stage lung cancer. For such cases, wedge resection has been widely recognized as a primary and effective treatment modality in numerous studies.

While our investigation predominantly focused on short‐term efficacy, we understood the criticality of long‐term follow‐up data for a holistic evaluation of surgical outcomes. Consequently, we are planning to conduct extensive long‐term follow‐up studies. These studies will involve prolonged monitoring of patients to examine key parameters such as survival rates, recurrence rates, and overall quality of life post‐treatment. In summary, our findings not only reaffirmed the safety of our surgical method but also established a robust foundation for future research and clinical practices in the realm of treating very early‐stage NSCLC.

## AUTHOR CONTRIBUTIONS

Yan Zhao: Conception, writing, statistical analysis, revision and data collection. Bin You: Conception and revision. Hui Li: Conception, revision and overall responsibility.

## CONFLICT OF INTEREST STATEMENT

The authors declare there are no conflicts of interest.

## Data Availability

Data can be available from the corresponding author upon reasonable request.
